# Novel Resistance Training Approach to Monitoring the Volume in Older Adults: The Role of Movement Velocity

**DOI:** 10.3390/ijerph17207557

**Published:** 2020-10-17

**Authors:** Diogo L. Marques, Henrique P. Neiva, Daniel A. Marinho, Mário C. Marques

**Affiliations:** 1 Department of Sport Sciences, University of Beira Interior, 6201-001 Covilhã, Portugal; diogo.marques@ubi.pt (D.L.M.); hpn@ubi.pt (H.P.N.); dmarinho@ubi.pt (D.A.M.); 2 Research Center in Sports Sciences, Health Sciences and Human Development, CIDESD, 6201-001 Covilhã, Portugal

**Keywords:** aging, functional capacity, low loads, low volume, strength, velocity loss

## Abstract

We analyzed the effects of velocity-monitored resistance training (RT) with a velocity loss of 20% on strength and functional capacity in institutionalized older adults. Thirty-nine participants (78.8 ± 6.7 years) were divided into a control group (CG; *n* = 20) or an RT group (*n* = 19). Over 10 weeks, the RT group performed two sessions per week, and the mean velocity of each repetition was monitored in the leg-press and chest-press exercises at 40–65% of one-repetition maximum (1RM). The set ended when the participants reached a velocity loss of 20%. The CG maintained their daily routine. At pre- and post-test, both groups were assessed in the 1RM leg-press, 1RM chest-press, handgrip strength, medicine ball throw (MBT), walking speed, and sit-to-stand (STS). At baseline, we did not find significant differences between groups. After 10 weeks, we observed significant differences (*p* < 0.001–0.01) between groups in the 1RM leg-press, 1RM chest-press, MBT-1 kg, and STS. The RT group performed a total number of repetitions of 437.6 ± 66.1 in the leg-press and 296.4 ± 78.9 in the chest-press. Our results demonstrate that velocity loss effectively prescribes the volume in older adults and that a threshold of 20% improves strength-related variables in this population.

## 1. Introduction

A significant challenge for public and private health services is to preserve functional capacity as people get older [1,2]. The progressive loss of skeletal muscle mass and strength, described as sarcopenia, contributes to a decrease in the capacity to generate force rapidly, leading to an increase in the incidence of falls and consequent bone fractures [3]. These common and devastating events in older populations are intrinsically related to institutionalization, morbidity, and mortality [3]. Therefore, reversing the deleterious effects of aging through effective evidence-based intervention programs must be considered a priority of the healthcare systems worldwide [1,2].

Resistance training (RT) is considered an effective method to improve strength and counteract age-related declines in older adults [4,5]. From a geriatric perspective, the manipulation of intensity (load) and volume (sets × repetitions) is essential to maximize strength gains, prevent injuries, and dropouts [5,6]. Evidence suggests that both low-to-moderate loads (<70% of one-repetition maximum [1RM]) and high loads (≥70% 1RM) are significant to improving muscle strength and functional capacity in older adults [5,6,7,8,9]. On the one hand, when using low-to-moderate loads, high movement velocities seem to be more effective than low velocities in increasing 1RM strength and functional capacity in older adults [10,11]. On the other hand, although high loads are also useful for improving strength and psychosocial well-being in older adults, they might be problematic for those with musculoskeletal impairments and for naïve RT practitioners [5]. Therefore, a low-load RT approach with high movement velocities might be a suitable strategy for older adults in order to improve 1RM strength and functional capacity, at least during the early phase of RT [5,6,10,11].

The literature is inconsistent and inconclusive regarding the optimal RT volume in older adults [12,13]. Both low and high volumes (i.e., one vs. three sets) seem to be equally useful for inducing strength adaptations in the short-term [12,13,14], yet more sets and repetitions appear to be required to increase 1RM strength in the long term [12,13,14]. Nevertheless, several studies already observed that higher volumes do not provide additional strength gains than lower volumes in older adults [5,12,14,15,16]. It is also important to note that when older adults perform a high number of repetitions per set closely to concentric failure, there is a higher acute cardiovascular, metabolic, and neuromuscular stress than for a low volume, which might be harmful in this population [17,18,19]. Therefore, considering that no consensus exists regarding the optimal training volume in older adults, alternative approaches must be evaluated.

Velocity-monitored RT is an effective strategy for improving physical performance and controlling the training load in trained young adults [20,21,22,23]. Using this method, coaches and practitioners can monitor the degree of fatigue and individualize the training volume by controlling the velocity loss during the sets [20,21,22,23,24]. Instead of a fixed, predetermined number of repetitions per set, the participants perform the repetitions until reaching a velocity loss threshold (e.g., 20%) [20,21,22,23]. Studies with trained young adults showed that a velocity loss lower or equal to 20% resulted in lower repetitions per set, and lower acute metabolic, hormonal, and mechanical fatigue than a velocity loss higher than 20% did [21,24]. Besides, in the long term, a velocity loss lower or equal to 20% promotes similar or even higher strength gains than a velocity loss higher than 20% does [22,23]. Thus, a velocity loss of around 20% seems to be enough to induce strength adaptations in trained young adults [22,23]. However, to date, no research has analyzed the effects of monitoring velocity loss during RT interventions in older adults. Considering that older individuals might benefit from one of three things: high loads, high effort, or high velocity [25], a combination of low loads and high movement velocities while monitoring velocity loss might probably be a more practical and safe approach in this population. This novel procedure would allow senior coaches and researchers to individualize the level of effort, avoid the adverse effects of fatigue, and eventually optimize the training stimulus [20,21,24].

Therefore, the purpose of the current research was to analyze the effects of velocity-monitored RT with a velocity loss of 20% in each set on strength and functional capacity in institutionalized older adults. Considering that older individuals exhibit a degree of adaptation to RT comparable to that of younger adults due to their neuromuscular plasticity [4,26,27], we hypothesized that a velocity loss of 20% would be a sufficient stimulus for enhancing muscle strength and functional capacity in this population. Moreover, we also hypothesized that performing a lower total number of repetitions than previously reported in high-velocity RT interventions with older people would be enough to increase 1RM strength.

## 2. Materials and Methods

### 2.1. Study Design

**Figure 1:**
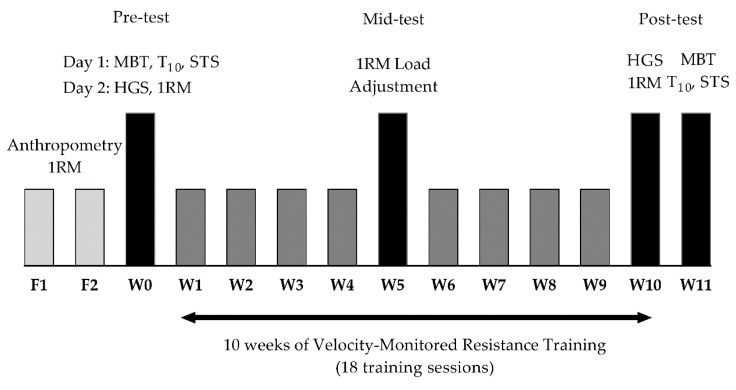
Study design; Abbreviations: 1RM: one-repetition maximum; F1: week 1 of familiarization; F2: week 2 of familiarization; HGS: handgrip strength; MBT: medicine ball throw; STS: five-repetition sit-to-stand; T_10_: 10 m walking speed; W: week.

This study was a nonblinded, nonrandomized controlled trial. Forty-five older adults living in community-dwelling centers were divided into an RT group or a control group (CG), based upon their perceived availability to attend the training sessions regularly. The participants reported their availability to the institutions’ geriatricians, who then communicated their decision to our research team. After that, we divided the participants between groups. Before the pretest, all participants underwent a familiarization period of two weeks (two sessions p/week) to ensure a proper adaptation to the fitness health club facilities, coaches, and exercises. During this period, we measured the body mass, in kg, (TANITA BC-601, Japan) and height, in m (Portable stadiometer SECA, Germany). We also performed a first assessment of the 1RM in the horizontal leg-press and seated chest-press exercises. After the adaptation period, we conducted two testing sessions separated by 48 h rest. In session 1, we measured the seated medicine ball throw distance with 1- (MBT-1kg) and 3-kg (MBT-3kg) medicine balls, the 10 m walking speed time (T_10_), and the time in the five-repetition sit-to-stand (STS). In session 2, we measured the handgrip strength (HGS) and the 1RM in the horizontal leg-press and seated chest-press. Following the pretests, the RT group performed a 10-week velocity-monitored RT program with two sessions per week separated by 48 h rest. The CG maintained their regular daily routine, without any form of physical exercise. In week 5 (session 10), we performed a new assessment of the 1RM in both exercises to adjust the absolute loads in the RT group [28]. At post-test, we first assessed the handgrip strength and the 1RM in the leg-press and chest-press in both groups because we aimed to analyze the performance on these tests immediately after the RT program (week 10, session 20). To avoid an excessive accumulation of fatigue that could impair the performance during the strength tests in the RT group, we decreased the number of sets in all exercises in session 19 (tapering strategy). After five days of rest (week 11), we assessed the MBT-1kg, MBT-3kg, T10, and STS in both groups. With five days of rest, we aimed to provide full recovery and increase the performance in tests that required high movement velocities. Figure 1 presents the schematic representation of the study design.

### 2.2. Participants

**Figure 2:**
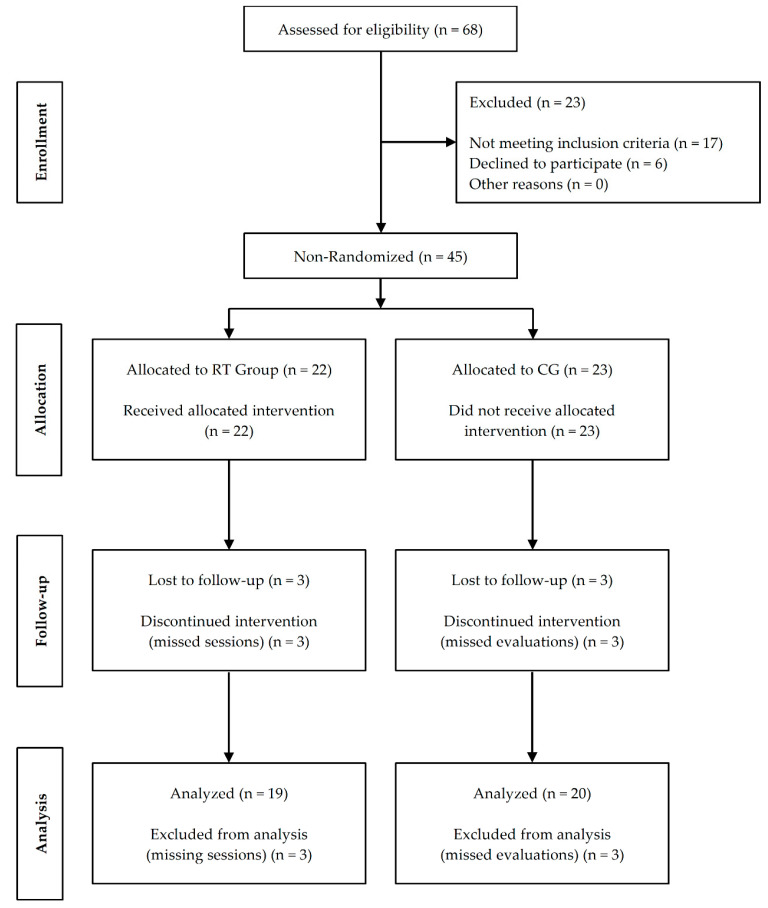
Study flow diagram; Abbreviations: CG: control group; RT: resistance training.

In collaboration with the geriatricians of several community-dwelling centers, we recruited institutionalized older adults to participate in this study. Inclusion criteria were age ≥ 65 years old, male and female, able to walk 10 m, independently stand up from a chair, with a willingness to participate in the study and collaborate with the researchers. Exclusion criteria were a simultaneous participation in another training program, severe cognitive impairment, cardiovascular/respiratory disorders, musculoskeletal injuries in the previous three months, and terminal illness. After screening, 30 women and 15 men without previous RT experience were divided into an RT group (*n* = 22) or a control group (CG; *n* = 23). From these, we excluded six participants due to the absence of training sessions and evaluations. Thus, 39 participants remained for the final analysis (Figure 2). Table 1 presents the characteristics of the participants at baseline. All participants received detailed information regarding the procedures and signed a written informed consent. The Ethical Committee of the University of Beira Interior (code: CE-UBI-Pj-2019-019) approved this study. The experimental procedures followed the recommendations of the Declaration of Helsinki.

### 2.3. Sample Size

To detect a final difference between groups of 20 kg in the 1RM leg-press [8] with a baseline SD of 21.91 kg and using an alpha of 5%, a sample size of 24 participants was needed to obtain a power of 80%. A dropout rate of 20% was also considered. The calculations were performed using a Microsoft Office Excel^®^ spreadsheet [29].

### 2.4. Outcome Measures

#### 2.4.1. One-Repetition Maximum Leg-Press and Chest-Press

All participants were assessed in two variable resistance machine exercises: horizontal leg-press (Leg-Press G3, Matrix, USA) and seated chest-press (Chest-Press G3, Matrix, USA). For the leg-press, the participants had to sit on the bench (lower back in contact with the machine), bend the knees at 90°, and place the feet shoulder-width apart on the platform. On command, they had to fully extend their legs, as fast and forcefully as possible, and slowly return to the initial position. In the chest-press, the participants had to sit on the bench, abduct the shoulders, flex the elbows at 90°, grab the handles with a full grip, and maintain the wrists in a neutral position. Then, we instructed them to perform a purely concentric action, as fast and forcefully as possible, and slowly return to the initial position. In the leg-press, we controlled the eccentric phase by standing alongside the participants and placing the hands on the platform handle. In the chest-press, we were behind the participants and placed the hands on the machine’s arms to control the descending phase. The general warm-up consisted of 10 min walking on a treadmill (2–4 km/h) or pedaling on a stationary bicycle (50–70 rpm with resistance levels varying between 1–5). The specific warm-up consisted of two sets (the first set of 5–10 repetitions at 40–60% of the perceived maximum load, followed by a 1 min rest, and the second set of 3–5 repetitions at 60–80% of the perceived maximum load). After that, 3–5 single attempts to reach the 1RM were conceded, with a 3–5 min rest between each maximal attempt. The procedures were already described elsewhere [17]. For the leg-press, the coefficient of variation (CV) was 2.83%, and the intraclass correlation coefficient (ICC) was 0.99 (95% confidence interval, CI: 0.98–0.99). For the chest-press, the CV was 3.55%, and the ICC was 0.99 (CI: 0.98–0.99).

#### 2.4.2. Handgrip Strength

The participants were seated on an armless chair (0.49 cm) in an erect position, with a 90° hip, knee, and elbow flexion position [17]. They exerted a maximal grip in both hands after instruction, using an adjustable portable digital hand dynamometer (Saehan, Model DHD-1) connected by USB to a personal computer. Three measures (~3 s) to the nearest 0.1 kg were performed with both hands, with a 1 min rest between each attempt. The three measures with both hands were averaged to calculate the absolute HGS. The CV was 3.54% in the left hand, and the ICC was 0.98 (CI: 0.98–0.99), while in the right hand the CV was 3.00%, and the ICC was 0.98 (CI: 0.98–0.99).

#### 2.4.3. Seated Medicine Ball Throw

Seated on an armless chair (0.49 cm) with the back straight and the medicine ball held in front of the chest, the participants had to throw the ball as far and fast as possible after instruction [17]. They performed three attempts with 1- and 3-kg medicine balls, with a 1 min rest between each attempt. The throwing distance was measured to the nearest 0.1 cm from the chest to where the ball landed, using a flexible tape. The best result was analyzed. For the 1-kg ball, the CV was 3.17%, and the ICC was 0.97 (CI: 0.96–0.98). For the 3-kg ball, the CV was 2.46%, and the ICC was 0.98 (CI: 0.96–0.98).

#### 2.4.4. 10 m Walking Speed

The walking speed time was recorded in an indoor wooden track. We instructed the participants to start one meter behind the starting line and finish one meter after the 10 m, to attenuate the acceleration and deceleration phases. After instruction, the participants walked over 10 m linearly as fast as possible, without running [7]. For safety, a coach walked alongside each participant while performing the test. The time was measured to the nearest 0.01 s using pairs of photoelectric cells (Race Time Kit 2, Microgate, Bolzano, Italy) attached to tripods, raised to a height of 0.5 m, and placed in pairs (0 and 10 m). Three trials separated by a 3 min rest were recorded, and the best time was analyzed. The mean velocity (MV) in T_10_ (T_10_-MV) was calculated by dividing the distance by the time (m·s^−1^). The CV was 2.98%, and the ICC was 0.95 (CI: 0.92–0.96).

#### 2.4.5. Five-Repetition Sit-To-Stand

The participants had to sit on an armless chair (0.49 cm) with the back straight and the arms crossed over the chest. After instruction, the participants stood up and sat down as fast as possible five times [30]. During the test, a coach stood alongside the participants to verbally encourage them and guarantee safety during the ascending and descending phases. The time was measured to the nearest 0.01 s using a digital stopwatch (Casio HS-3V-1R, Tokyo, Japan). Two trials, separated by 2 min, were conceded, and the best one was analyzed. The STS-MV (m·s^−1^) and the STS mean power (STS-MP) (Watts, W), were calculated using the equations proposed by Alcazar et al. [30]. The CV was 2.64%, while the ICC was 0.94 (CI: 0.91–0.96).

### 2.5. Resistance Training Program

All training sessions were supervised by an experienced researcher and three specialist senior coaches to ensure safety and the proper execution of all the exercises. The sessions lasted 45 min and were performed in a fitness health club, at the same time (2:00–3:00 pm), with a room temperature of 22–24 °C. After a general warm-up of 10 min walking on a treadmill (2–4 km/h) or pedaling on a stationary bicycle (50–70 rpm; resistance levels: 1–5), the participants performed the following exercises: horizontal leg-press; seated chest-press; MBT; chair squats with a weight-vest. Between sets and exercises, they rested for 2–3 min. The cool-down consisted of 5 min walking or pedaling at low intensity. For the leg-press and chest-press, the relative loads progressed from 40–65% 1RM [5]. The training volume consisted of 2–3 sets with a velocity loss of 20%. The sets ended when the participants reached the 20% threshold [23]. We verbally encouraged the participants to perform the concentric phase as fast and forcefully as possible and slowly return to the initial position. In the leg-press, coaches controlled the eccentric phase by standing alongside the participants and placing their hands on the platform handle. In the chest-press, coaches were behind the participants and controlled the eccentric phase by placing their hands on the machine’s arms. Table 2 shows the characteristics of the training program.

### 2.6. Data Collection

**Figure 3:**
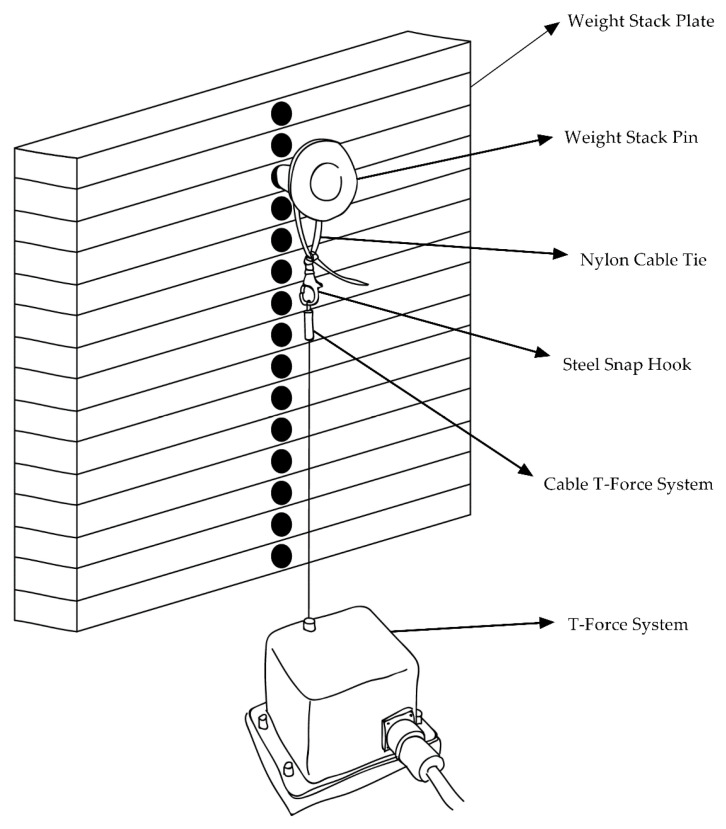
Illustration of the connection between the T-Force System and the resistance machines.

The MV (i.e., the average velocity from the start of the concentric phase until the weight stack plate reached the maximum height) of each repetition was recorded in real time using a linear velocity transducer (T-Force System, Ergotech, Murcia, Spain) [31]. The T-Force collects data at a sampling frequency of 1000 Hz and is a valid and reliable device to measure kinetic and kinematic variables during RT [32]. We connected the T-Force to the resistance machines by attaching a steel snap hook with a nylon cable tie to the T-Force cable extension. Following this, we attached the nylon cable tie to the weight stack pin that fixed the load (Figure 3). The load and the T-Force cable extension were simultaneously displaced in a vertical direction, allowing the measurement of MV. A custom software (T-Force v2.36) displayed the data in real time. In every session, we analyzed the following variables: total repetitions (sum of all completed repetitions), repetitions per set (average of repetitions performed in each set), fastest MV (maximum value of MV attained), average MV (average MV of all repetitions), and velocity loss (average of the percent change from the fastest to the slowest repetition in each set). In the software, we selected the option to identify the fastest MV in the first three repetitions. In the leg-press, the fastest MV was attained, on average, in repetition 2.6 ± 0.5, while in the chest-press it was attained in repetition 2.2 ± 0.3.

### 2.7. Statistical Analysis

Data are presented as mean ± SD unless otherwise indicated. The normality and homogeneity of variances were calculated and confirmed using the Shapiro–Wilk and Levene tests, respectively. The ICC (95% CI) was calculated using a two-way random effect, absolute agreement, single rater/measurement model (ICC_2,1_) [33], while the CV was calculated as (SD/mean) × 100. An independent-samples *t*-test analyzed the differences between groups at baseline and between the variables collected during the leg-press and chest-press exercises. A mixed design 2 × 2 factorial analysis of variance (ANOVA) analyzed the differences between groups (RT group, CG) and time (pretest, post-test) for all variables. Paired samples t-tests compared the differences within groups from pre- to post-test. A repeated-measures ANOVA (within subject-factor: time 4 levels) with post hoc Bonferroni adjustments analyzed the differences in the number of repetitions per set and the fastest MV attained against the same relative load (e.g., fastest MV in session 1 at 40% 1RM vs. fastest MV in session 2 at 40% 1RM vs. fastest MV in session 3 at 40% 1RM vs. fastest MV in session 4 at 40% 1RM). The percentage change was calculated with a 90% CI. The effect size (ES) between and within groups was calculated using Hedge’s *g* formula [34]. The ES was interpreted as follows: trivial, 0.0–0.2; small, 0.2–0.6; moderate, 0.6–1.2; large, 1.2–2.0; very large, 2.0–4.0; extremely large, >4.0 [35]. The alpha level was set at *p* < 0.05. Statistical analyses were performed in Microsoft Office Excel^®^ (Microsoft Inc., Redmond, WA, USA) and SPSS v26 (SPSS Inc., Chicago, IL, USA). The data were plotted in GraphPad Prism v7 (GraphPad Inc., San Diego, CA, USA).

## 3. Results

At baseline, we did not observe significant differences between groups in any of the analyzed variables. Changes from pre- to post-test are presented in Table 3. After 10 weeks, significant differences between groups were observed in the 1RM leg-press, 1RM chest-press, MBT-1kg, STS, STS-MV, and STS-MP. We observed significant gains in 1RM leg-press, 1RM chest-press, MBT-1kg, STS, STS-MV, and STS-MP in the RT group. In CG, we found a significant decrease in T_10_-MV.

Table 4 shows a general description of the acute RT variables in the leg-press and chest-press. The total repetitions and the number of repetitions per set in the leg-press were significantly higher than in the chest-press. The fastest and average MV values in the leg-press were higher than in the chest-press. We observed significant differences between the fastest MV and the average MV in the leg-press (*p* < 0.001; ES = 0.83) and chest-press (*p* < 0.001; ES = 0.73). The velocity loss in the leg-press was lower than in the chest-press.

**Figure 4:**
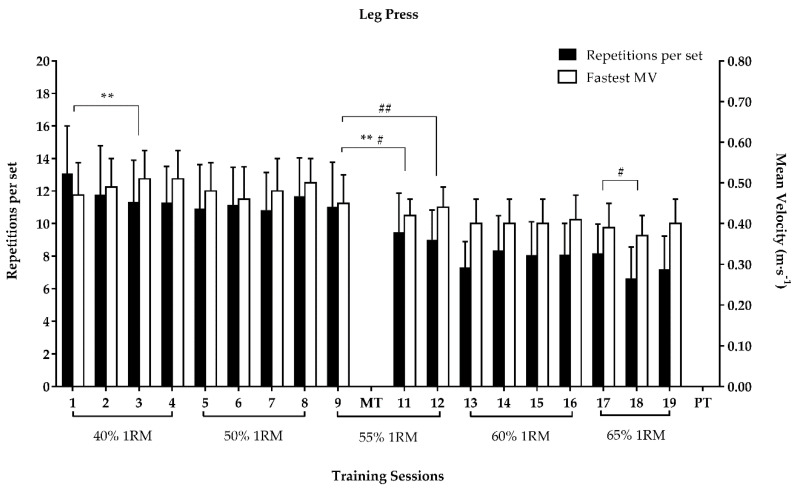
Repetitions per set and fastest MV (mean ± SD) in the leg-press exercise throughout the RT program; 1RM: one-repetition maximum; MT: 1RM mid-test load adjustment; MV: mean velocity; PT: post-test; ** *p*-value < 0.01 for the fastest MV; # *p*-value < 0.05 for the number of repetitions per set; ## *p*-value < 0.01 for the number of repetitions per set.

**Figure 5:**
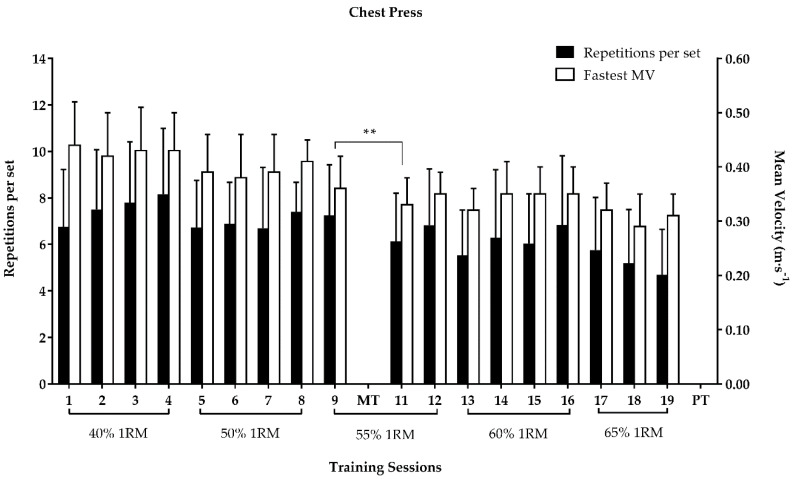
Repetitions per set and fastest MV (mean ± SD) in the chest-press exercise throughout the RT program; 1RM: one-repetition maximum; MT: 1RM mid-test load adjustment; MV: mean velocity; PT: post-test; ** *p*-value < 0.01 for the fastest MV.

The repetitions per set performed in the leg-press at 55% 1RM significantly decreased from session 9 to sessions 11 and 12 (Figure 4). The fastest MV in the leg-press at 40% 1RM significantly increased from session 1 to 3, while at 55% 1RM it significantly decreased from session 9 to 11 (Figure 4). The fastest MV in the chest-press at 55% 1RM significantly decreased from session 9 to 11 (Figure 5).

## 4. Discussion

We analyzed the effects of velocity-monitored RT with a velocity loss of 20% on strength and functional capacity in institutionalized older women and men. The main finding was that a velocity loss of 20% was sufficient to increase strength and functional capacity in older adults, thus confirming our main hypothesis. Therefore, these data support velocity loss as an effective variable to prescribe the training volume in older adults. Our results also confirm our second hypothesis that performing a lower total number of repetitions than previously reported in high-velocity RT interventions with older people is enough to increase 1RM strength.

Although 1RM gains have been similar and, in some cases, higher than those reported in previous high-velocity RT studies with older adults [8,9,10,36,37,38,39,40,41,42], the total number of repetitions performed in the leg-press and chest-press was inferior compared to all studies. Based on the study duration, sessions per week, sets, and repetitions performed in only one exercise, a total number of repetitions between 600 and 1056 in the chest-press [10,11,36,37,39,41,42] and leg-press [8,9,10,11,36,37,38,39,40,41,42] was performed in these studies, which means ~50% more than the total repetitions performed in our study. Therefore, these results suggest that a low volume is as effective as a high volume for improving 1RM strength in older adults. A previous study with older adults corroborates this observation [15]. Participants who performed 50% of the possible maximal repetitions increased their 1RM strength gains to a similar extent as those that performed the repetitions until concentric failure did [15]. Thus, taken together, this evidence suggests that, with a low number of repetitions per set completed at a high movement velocity, it is possible to achieve similar strength gains in older adults when compared to a high number of repetitions per set. Despite the low number of total repetitions, one possible explanation for the 1RM strength gains might be associated with the use of high movement velocities, which seems to promote an increase in type II fast-twitch fibers in older adults after RT [26,43]. However, to our knowledge, most studies that assessed muscle fiber changes after RT using high movement velocities either applied a combination of low and high loads [26] or only high loads [43]. Thus, future studies should investigate the influence of high movement velocities against low loads on fast-twitch fiber changes in older adults. Another possible cause for the 1RM gains might be related to the use of velocity loss. Using this variable during each RT session, we could control the degree of fatigue and individualize the training volume, which might have contributed to optimizing the training stimulus and consequently enhancing the 1RM strength in the leg-press and chest-press.

Our results demonstrated that, despite the prescribed magnitude of velocity loss had been identical in both exercises, the number of repetitions per set was significantly higher in the leg-press than in the chest-press (~3 repetitions more). These data suggest that the upper muscles fatigue faster than the lower muscles in older adults when matching the same velocity loss. Our study supported this evidence by the significantly higher percentage of velocity loss observed in the chest-press than in the leg-press. These differences can be explained by the smaller muscle groups involved during upper body exercises (e.g., bench press) compared to lower body exercises (e.g., squat). Besides, the higher presence of fast-twitch fibers in the upper musculature causes a higher degree of fatigue [20,24]. Therefore, as observed in a study with trained young adults, to equalize the number of repetitions per set, the magnitude of the velocity loss must be different (at least by 5%) between the lower and upper body exercises [24]. However, these results were only observed in younger populations, which means that this evidence remains to be explored in older adults.

In the leg-press, from sessions 1 to 3 we observed a significant increase in MV at 40% 1RM. Considering that an increase in MV against the same weight is an indicator of performance improvement [23], our participants’ strength increased, possibly after one week. In a study with older adults that evaluated changes in strength during RT, the authors observed a significant increase of 10% in the maximal force after repeated isometric contractions over only two days [27]. Similarly, some studies observed significant increases in 1RM after 5–6 weeks of RT in older adults [28,44]. In our study, the significant decreases in MV from session 9 to 11 in both resistance exercises were influenced by an increase in the weight after the 1RM mid-test (load adjustment), which indicated a strength improvement after five weeks. Thus, taken together, these results reflect the early and rapid increases in muscle strength in older adults, which can be mainly attributed to neural adaptations [26,27].

After 10 weeks, we did not observe any change in the HGS in the RT group. Although the HGS is a strong predictor of mortality and an indicator of general strength, its sensitivity is questionable in relation to detecting physical performance changes in older adults after RT interventions [45]. Thus, future studies should analyze the underlying mechanisms for nonsignificant changes in the HGS after RT in this population. Considering that we only included exercises for the chest and the quadriceps, future studies should also include exercises targeting the forearm muscles to analyze their influence on the HGS.

At post-test, we observed significant gains in the MBT-1kg, while in the MBT-3kg we found a nonsignificant increase. These differences can be justified because only the MBT-1kg was included as part of the RT program. Indeed, when the same medicine ball weight is used both in the test and the intervention, significant gains tend to occur [7,8,46]. Conversely, when the MBT is not included in the RT program, the findings are less conclusive about the transference effects of RT on this parameter. In a study that analyzed the effects of 12 weeks of high-velocity RT on the MBT-3kg distance in older individuals, nonsignificant gains of 3% were observed in the group that performed the RT in pneumatic machines, and a significant gain of 6% was observed in the group that performed the RT in plate-loaded machines [47]. In that study, the participants performed three sets of 8–10 repetitions in six upper body exercises. Considering that our participants only used the chest-press exercise and performed a lower number of repetitions than in that study, more exercises should be included and more repetitions performed, possibly to enhance the MBT-3kg distance. Future studies should include exercises targeting the shoulder flexors and elbow extensors to analyze their transference effect on the ball throwing distance with heavier weights in older adults.

Despite the nonsignificant improvements in T_10_, our results found a relevant aspect. In the RT group, the T_10_-MV increased, while in the CG it significantly decreased. These results suggest the loss of walking speed during aging and reinforce the importance for older adults to engage in RT [8]. Studies observed significant gains in T_10_ (−18% to −6%) after high-velocity RT programs with older adults [7,8,9,40]. On average, the total repetitions varied between 576 and 864. More than one lower body exercise was used in three of them: leg-press, leg-extension, and leg-curl [8,9,40]. Thus, increasing the walking speed in older adults may require more volume and exercises targeting both the quadriceps and the hamstring muscles. However, future studies are warranted to confirm this hypothesis.

In our study, we observed significant decreases in the STS time. This result agrees with previous findings, in which significant gains from −15% to −11.8% were observed after high-velocity RT with older adults [36,37,48]. Of these, only one study prescribed a total number of repetitions per exercise lower than ours (~312 repetitions) [48]. However, given that the leg-press, knee-extension, and leg-curl were included, the participants performed ~936 repetitions on average. In the studies of Henwood and Taaffe [37], and Balachandran et al. [36], three and two lower-body exercises were used, resulting in approximately 1800 and 3168 repetitions, respectively. Thus, comparing these numbers to ours, we present an efficient and effective strategy to improve the ability to rise from a chair and enhance older adults’ functional capacity.

This study presents some limitations. A larger sample size would allow us to generalize the results and reduce the probability of a type II error. Moreover, an additional experimental group could give us important insights into the effects of different velocity loss thresholds on older adults’ strength and functional capacity. Including resistance exercises targeting the forearm muscles could be important to analyze their effects on the HGS. Therefore, future velocity-monitored RT interventions with older adults should include larger sample sizes, more experimental groups, and additional exercises targeting the forearm muscles.

In summary, our data suggest that monitoring the velocity loss during RT is an efficient and effective strategy to prescribe the training volume in older adults and to increase muscle strength and functional capacity in this population.

## 5. Conclusions

The current research presents a novel RT approach to prescribe the volume in older adults by monitoring each set’s velocity loss. The training method presented here opens a new possibility for coaches and clinicians to adopt an individualized intervention and optimize muscular adaptations during RT with older adults. In practical terms, two RT sessions per week with a velocity loss of 20% (i.e., 2–3 sets of ~10 and 7 repetitions per set in the leg-press and chest-press, respectively) and relative loads progressing from 40–65% 1RM seem to be enough to induce muscle strength adaptations and improve functional capacity in older adults aged between 70 and 90 years.

## Figures and Tables

**Table 1:** Participants’ characteristics at baseline.

Variable	RT Group (12 Women; 7 Men)	CG (14 Women; 6 Men)	*p*-Value
Age (years)	78.6 ± 7.6 (range: 69 to 92)	79.0 ± 6.0 (range: 70 to 89)	0.85
Body mass (kg)	70.4 ± 14.3	70.3 ± 12.6	0.98
Height (m)	1.55 ± 0.11	1.57 ± 0.09	0.68
BMI (kg/m^2^)	29.3 ± 5.4	28.6 ± 4.0	0.66
MMSE	24.3 ± 2.3	24.5 ± 1.8	0.78

Notes: Data are presented as mean ± SD. Abbreviations: BMI: body mass index; CG: control group; MMSE: minimental state examination; RT: resistance training group.

**Table 2:** Resistance training program.

	Week 1		Week 2		Week 3		Week 4		Week 5	
Exercises	TS1	TS2	TS3	TS4	TS5	TS6	TS7	TS8	TS9	TS10
LP & CP (S × VL [%])	2 × 20%	2 × 20%	3 × 20%	3 × 20%	2 × 20%	2 × 20%	3 × 20%	3 × 20%	2 × 20%	1RM Mid-test Load Adjustment
1RM (%)	40%	40%	40%	40%	50%	50%	50%	50%	55%	
Chair-Squat (S × R × kg)	1 × 10 × 3	1 × 10 × 3	2 × 10 × 3	2 × 10 × 3	2 × 10 × 3	2 × 10 × 3	3 × 10 × 3	3 × 10 × 3	3 × 10 × 3	
MBT (S × R × kg)	1 × 10 × 1	1 × 10 × 1	2 × 10 × 1	2 × 10 × 1	2 × 10 × 1	2 × 10 × 1	3 × 10 × 1	3 × 10 × 1	3 × 10 × 1	
	**Week 6**		**Week 7**		**Week 8**		**Week 9**		**Week 10**	
**Exercises**	**TS11**	**TS12**	**TS13**	**TS14**	**TS15**	**TS16**	**TS17**	**TS18**	**TS19**	**TS20**
LP & CP (S × VL [%])	2 × 20%	2 × 20%	3 × 20%	3 × 20%	2 × 20%	2 × 20%	3 × 20%	3 × 20%	2 × 20%	1RM Post-test
1RM (%)	55%	55%	60%	60%	60%	60%	65%	65%	65%	
Chair-Squat (S × R × kg)	4 × 8 × 3	4 × 8 × 3	4 × 8 × 3	4 × 8 × 3	4 × 8 × 3	4 × 8 × 3	4 × 8 × 3	4 × 8 × 3	2 × 8 × 3	
MBT (S × R × kg)	4 × 8 × 1	4 × 8 × 1	4 × 8 × 1	4 × 8 × 1	4 × 8 × 1	4 × 8 × 1	4 × 8 × 1	4 × 8 × 1	2 × 8 × 1	

Abbreviations: 1RM: one-repetition maximum; CP: chest-press; LP: leg-press; MBT: medicine ball throw; R: repetitions; S: sets; TS: training session; VL: velocity loss.

**Table 3:** Changes in strength-related variables from pre- to post-test in the resistance training group and CG (mean ± SD).

	RT Group				CG				RT vs. CG	
Variable	Pre-Test	Post-Test	Δ (90% CI)	ES	Pre-Test	Post-Test	Δ (90% CI)	ES	*p*	ES
1RM LP (kg)	74.79 ± 23.12	85.00 ± 22.79 ***	15.07 (11.77 to 18.37)	0.43	67.65 ± 20.5	67.15 ± 18.42	−0.26 (−1.81 to 1.29)	−0.02	<0.001	0.85
1RM CP (kg)	31.54 ± 13.15	39.26 ± 12.48 ***	31.35 (21.05 to 41.66)	0.58	30.10 ± 9.40	29.75 ± 8.58	−0.24 (−2.84 to 2.37)	−0.04	<0.001	0.87
HGS (kg)	25.20 ± 8.50	25.07 ± 8.01	−0.08 (−2.34 to 2.18)	−0.02	25.55 ± 7.19	25.15 ± 6.90	−1.08 (−3.74 to 1.59)	−0.05	>0.05	−0.01
MBT-1kg (m)	3.01 ± 0.60	3.24 ± 0.62 ***	8.09 (4.80 to 11.38)	0.37	2.91 ± 0.59	2.87 ± 0.64	−1.59 (−3.67 to 0.50)	−0.06	<0.001	0.57
MBT-3kg (m)	2.26 ± 0.43	2.31 ± 0.38	2.99 (0.04 to 5.94)	0.12	2.13 ± 0.37	2.17 ± 0.43	1.74 (−1.23 to 4.70)	0.10	>0.05	0.33
T_10_ (s)	6.17 ± 0.82	6.05 ± 0.94	−1.77 (−5.94 to 2.40)	−0.14	6.57 ± 0.99	6.69 ± 0.91	2.00 (0.46 to 3.55)	0.12	>0.05	−0.68
T_10_-MV (m·s^−1^)	1.65 ± 0.22	1.69 ± 0.25	3.04 (−1.40 to 7.47)	0.18	1.55 ± 0.22	1.52 ± 0.20 *	−1.81 (−3.24 to −0.38)	−0.15	>0.05	0.74
STS (s)	9.70 ± 1.22	8.56 ± 1.39 ***	−11.72 (−14.73 to −8.71)	−0.84	10.36 ± 1.10	10.38 ± 1.01	0.38 (−0.99 to 1.75)	0.02	<0.001	−1.48
STS-MV (m·s^−1^)	0.29 ± 0.07	0.33 ± 0.08 ***	13.93 (9.89 to 17.97)	0.51	0.28 ± 0.05	0.27 ± 0.05	−0.24 (−1.61 to 1.13)	−0.01	<0.001	0.78
STS-MP (W)	183.54 ± 73.66	211.12 ± 87.37 ***	14.86 (10.61 to 19.12)	0.33	173.93 ± 55.72	173.27 ± 56.80	−0.47 (−1.87 to 0.92)	−0.01	<0.001	0.51

Notes: * *p*-value < 0.05; *** *p*-value < 0.001; Abbreviations: Δ: percent change; 1RM: one-repetition maximum; CG: control group; CI: confidence interval; CP: chest-press; ES: effect size Hedge’s *g*; HGS: absolute handgrip strength; LP: leg-press; MBT: medicine ball throw; MP: mean power-output; MV: mean velocity; RT: resistance training; STS: five-repetition sit-to-stand; T_10_: 10 m walking speed.

**Table 4:** Overall description of the acute training variables in the leg-press and chest-press exercises.

	Leg-Press	Chest-Press	*p*	Effect Size	
Variable	Mean (95% CI)	Mean (95% CI)	Between	*g*	Magnitude
Total repetitions	437.63 (407.89 to 467.37)	296.37 (260.89 to 331.84)	<0.001	1.90	Large
Repetitions per set	9.75 (8.44 to 11.06)	6.58 (5.48 to 7.68)	<0.001	1.15	Moderate
Fastest MV (m·s^−1^)	0.44 (0.41 to 0.48)	0.37 (0.33 to 0.40)	<0.001	0.97	Moderate
Average MV (m·s^−1^)	0.38 (0.35 to 0.41) ^a^	0.31 (0.28 to 0.35) ^b^	<0.001	0.98	Moderate
Velocity loss (%)	22.87 (22.16 to 23.59)	23.77 (22.80 to 24.73)	<0.001	−0.46	Small

Notes: ^a^ Denotes a significant difference (*p* < 0.001) between the fastest MV and the average MV in the leg-press exercise; ^b^ Denotes a significant difference (*p* < 0.001) between the fastest MV and the average MV in the chest-press exercise; Abbreviations: CI: confidence interval; ES: effect size Hedge’s *g*; MV: mean velocity.
